# Climate-Smart Livestock Systems: An Assessment of Carbon Stocks and GHG Emissions in Nicaragua

**DOI:** 10.1371/journal.pone.0167949

**Published:** 2016-12-28

**Authors:** Lucía Gaitán, Peter Läderach, Sophie Graefe, Idupulapati Rao, Rein van der Hoek

**Affiliations:** 1 International Center for Tropical Agriculture (CIAT), Managua, Nicaragua; 2 Tropical Silviculture and Forest Ecology, Georg-August-Universität Göttingen, Germany; 3 International Center for Tropical Agriculture (CIAT), Cali, Colombia; University of Delhi, INDIA

## Abstract

Livestock systems in the tropics can contribute to mitigate climate change by reducing greenhouse gas (GHG) emissions and increasing carbon accumulation. We quantified C stocks and GHG emissions of 30 dual-purpose cattle farms in Nicaragua using farm inventories and lifecycle analysis. Trees in silvo-pastoral systems were the main C stock above-ground (16–24 Mg ha^-1^), compared with adjacent secondary forests (43 Mg C ha^-1^). We estimated that methane from enteric fermentation contributed 1.6 kg CO_2_-eq., and nitrous oxide from excreta 0.4 kg CO_2_-eq. per kg of milk produced. Seven farms that we classified as climate-smart agriculture (CSA) out of 16 farms had highest milk yields (6.2 kg cow^-1^day^-1^) and lowest emissions (1.7 kg CO_2_-eq. per kg milk produced). Livestock on these farms had higher-quality diets, especially during the dry season, and manure was managed better. Increasing the numbers of CSA farms and improving CSA technology will require better enabling policy and incentives such as payments for ecosystem services.

## Introduction

Livestock production occupies two-thirds (34 Mkm^2^) of the world´s agricultural land (49 Mkm^2^) for production of animal feed (grazed pastures, 80%, and feed crop, 20%), while a quarter (3.5 Mkm^2^) of the crop area (15.2 Mkm^2^) produces animal feed [1–3]. In the Brazilian Amazon region, which represents 37% of Brazilian herds, cattle ranching is intertwined with deforestation, which globally was the largest region contributing to deforestation during 1990–2010 [4]. Deforestation in Brazil releases 590 t of CO_2_-equivalent (CO_2_-eq.) for each hectare cleared [5]. Moreover, in Latin America, an estimated 2 Mkm^2^ of grazing land is severely degraded [6] with low forage availability, reduced vegetative cover and lost soil fertility.

Global demand for livestock products, principally milk and meat, is expected to double by 2050, particularly in developing countries [7, 8]. Livestock production is responsible for over 50% of greenhouse gas (GHG) emissions from agriculture [9–11], accounting for 7.1 billion t CO_2_-eq. yr^-1^. Globally, emissions from the livestock sector represent 14.5% of anthropogenic emissions [12], with beef and milk cattle accounting for 41% and 21%, respectively [13]. Recent analysis of beef production showed that some grass-fed beef systems have lower climate impact than feedlot systems [14].

To address these challenges, agriculture must use resources more efficiently and become more resilient to climate change [15]. Climate-smart agriculture (CSA) aims to improve global food security, while promoting adaptation to climate change and contribute to its mitigation [16]. With respect to CSA, livestock systems in the tropics can contribute to mitigate climate change by reducing GHG emissions and increasing carbon accumulation [17, 18, 16, 13]. Well-managed improved forages can accumulate carbon (C) in the soil at amounts second only to forest [19]. Moreover, replacing naturalized grasses with varieties of higher quality and digestibility reduces the amount of methane emitted per unit of milk or meat produced [20, 21]. The vegetative cover of well-managed pastures enhances ecosystem services by controlling soil erosion and restoring land through increased soil organic matter and better microclimate [22].

Brachiaria grasses are the most widely used forages for livestock in the tropics [23]. *B*. *humidicola* releases biological nitrification inhibitors (BNIs) from its roots, which reduce nitrous oxide (N_2_O) emissions by inhibiting nitrification in the soil [24]. Silvopastoral systems combine forage grasses with trees and shrubs, improving animal nutrition and generating co-benefits like improved soil fertility and increased C accumulation [25]. In Latin America and the Caribbean (LAC), well-managed silvopastoral systems with specific tree species accumulate larger amounts of carbon than secondary forest [26–29].

Improved forage-based systems contribute to climate change adaptation. Forage grasses and legumes that are resilient to stress, provide feed for livestock during drought or waterlogging [13]. Drought-adapted forage legumes in crop-livestock systems can provide high-quality feed in the dry season [30].

REDD (reducing emissions from deforestation and forest degradation) and PES (payment for ecosystem services) proposals offer incentives such as carbon credits to mitigate and adapt to climate change. Such proposals include policies that encourage storage of carbon in forests and on agricultural land [31]. One example is carbon insetting, the concept of “integrating carbon credit purchases into a company’s own supply chain” [32, 33]. Benefits are shared between the producer and the buyer and usually contribute simultaneously to climate change adaptation and mitigation.

REDD is exclusively for forests, while there are also schemes for carbon credits with cash crops like cocoa and coffee [34–36]. Although the livestock sector is agriculture’s major contributor to GHG emissions, it has no similar initiatives. A first step would be to devise systems of livestock production that reduce GHG emissions and increase the potential to accumulate C. A second step would be to devise a certification scheme for smallholders that would allow them to benefit from climate-smart production of livestock.

We aimed to quantify existing livestock production systems in terms of carbon stocks and GHG emissions. We also sought to identify CSA systems that might be used as the basis of PES schemes such as carbon credits. We further tested whether with good management, milk production can increase while GHG emissions decrease.

## Materials and Methods

The Nicacentro cooperative provided the permission for each location through the project “Competitive beef and dairy through sustainable intensification and specialized market access”, where CIAT was a partner.

We state clearly that no specific permissions were required for these locations/activities, because the study was part of the before mentioned project. We confirm that the field studies did not involve endangered or protected species

### Study site

**Fig 1:**
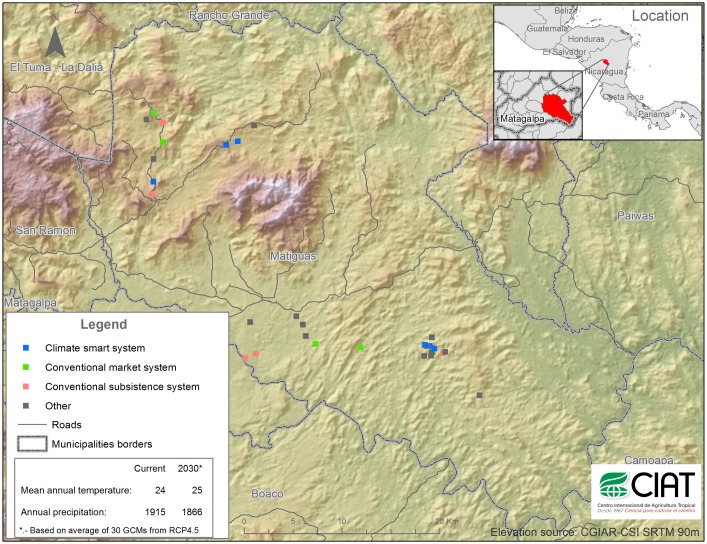
Study site at Matiguás, Nicaragua. Location of the 30 farms, of which 16 were classified as one of the three typologies: conventional subsistence, conventional market and climate smart. Reprinted from [39] under a CC BY license, with permission from [CGIAR-CSI], original copyright [2008].

The study was carried out in the municipality of Matiguás (85°42’N, 12°8’W, 270–680 m.a.s.l.), Department of Matagalpa, Nicaragua (Fig 1) during October–November, 2014. The area is classified as humid tropical forest [37] with mean annual temperature of 24°C and mean annual rainfall of 1915 mm. Precipitation is unimodal with a wet season May–December and a dry season January–April [38].

### Overall approach for farm selection to estimate C stock and GHG emissions

We selected 30 small- and medium-sized dairy farms that had two or more systems of land use: naturalized pasture without trees (NP), naturalized pasture with 20 or more trees ha^-1^ (NPT), improved pasture without trees (IP), improved pasture with 20 or more trees ha^-1^ (IPT), and secondary forest (SF). Most farms typically included a mixture of these five land use types.

For each land use system, we performed an inventory of vegetation to estimate C stocks. Simultaneously, we conducted inventories of farmer management practices by means of semi-structured interviews. For every farm we recorded area per type of land use, herd size and composition, use of supplementary feeding, farm infrastructure and productivity, agrochemical use, and management and production of forages as cattle feed. We then estimated GHG emissions using these data and analyzed the interactions between land-use types, management practices, GHG emissions, and milk yield. From this analysis 16 farms corresponded to one of the three categories of livestock production systems: subsistence system, conventional market system, and climate-smart system. The remaining 14 farms did not fit into one of the three categories since there was not a clear interaction between the variables analyzed.

The farmers questionnaire to calculate GHG emissions was divided into four sections. The first section related to social capital inquiring about technical assistance, capacity building, strategies to improve productivity and access to public services. The second section related to natural capital, such as farm area, land use types and durations, use of shade trees, and presence of water bodies. The third section focused on physical capital, including information on transportation, grazing management, farm infrastructure, and use of fossil fuel. The fourth section dealt with productive capital, with questions related to herd inventory, breeds, agro-chemical inputs, manure management, supplementary feeding, milking (frequency, production), and reproduction.

### Quantification of carbon stocks

We estimated the above-ground biomass of wood, herbs and litter. We inventoried all trees with diameter >10 cm by species and diameter at breast height (DBH). We applied allometric equations to estimate wood density and life zone for each species to convert DBH into tree biomass [37, 38, 40, 41]. We extracted density from the global data base of wood density [42] and converted biomass to C using the factor 0.47 [43].

We sampled the trees in SF in plots of 50 x 20 m, recording every tree with DBH > 10 cm [44]. Inside the main sampling plot, we established 50 cm x 50 cm subplots to measure litter biomass. In the pasture-based systems, we used plots of 71 x 71 m to ensure that all trees were included.

We used the BOTANAL procedure [45, 46] to determine the biomass of the grasses (*B*. *brizantha* ‘Toledo’ and ‘Marandu’) in IP and *Paspalum notatum* in NP. This procedure is based on visual estimation of yield and species abundance, calibrated with selected harvest samples, to calculate forage dry matter and botanical composition of a pasture.

For soil organic carbon (SOC), we used data from [38], which covered similar land-use systems in Matiguás.

### Estimation of GHG emissions

We estimated GHG emissions using life cycle analysis (LCA), which assesses the environmental impacts and resource use of different production systems [47]. We report estimated GHG emissions in kg CO_2_-eq. (kg fat-and-protein-corrected milk)^-1^ (FPCM) and (kg live weight gain)^-1^ (LWG). Input data for LCA was acquired through the previously described questionnaire. [48]. We included all relevant on- and off-farm activities to assess the emissions of the final product at the farm gate. We estimated on-farm emissions from animal digestion, manure management, use of fertilizer and fossil fuel used in machinery. We included off-farm emissions of fossil fuel for transportation of farm inputs, and emissions originating from the manufacturing of farm inputs, such as herbicides, fertilizer and concentrates.

We calculated farm GHG emissions with a calculator tool developed by CATIE (Tropical Agricultural Research and Higher Education Center) based on the parameters and emission factors from the IPCC guidelines, [49] using TIER I to calculate methane emissions from manure, and N_2_O emissions from manure and waste management. For estimating methane (CH_4_) emissions from enteric fermentation, we combined emission factors developed by CATIE (TIER II) with default values from IPCC (TIER I) [50, 51]. TIER I refers to emissions based on default factors, whereas TIER II accounts for detail of herd composition, productivity and residue management [52]. Main input data included the geographic region, average temperature, TIER level, reproductive cycle (production period for milk) and global warming potential for CH_4_ and N_2_O according to IPCC (2006). Since CH_4_ emissions from enteric fermentation were based on TIER II factors, the input data was specific to type of cattle: lactating cow, dry cow, heifer 1–2 years, heifer > 2 years, calf and bull. Other input data included grazing category (extensive or intensive), manure management type, live weight of cattle (defined as the weight of the animal before slaughter), milk production, liveweight gain per animal and per hectare, calving rate, forage digestibility and protein content [48, 53]. The CH_4_ emissions in TIER II come from enteric fermentation and manure residues, N_2_O emissions from management of manure residues and fertilizers. The CO_2_ emissions in TIER I come from fossil fuels used in machinery, vehicles, and the production of herbicides, fertilizers and concentrates [43, 50]. Emission factors from input production were acquired based on the life cycle analysis software tool SIMAPRO 7.2. This software uses the Ecoinvent database containing emission factors from urea (fertilizer), glyphosate and 2-4-D (pesticide), and maize (concentrate).

We estimated emissions separately for the wet and dry season, because livestock are managed differently during those periods. Forage availability during the dry season reduces drastically, and crop residues and supplements such as mineral salts and locally available concentrates such as tree pods are commonly added to the diet. During the wet season pasture is the main feed source and milk yield is higher compared to the dry season.

GHG emission calculations were based on the survey results except the data on feed quality. The cooperative Nicacentro provided data on percentage dry matter, protein and metabolizable energy of each diet component, while the International Center for Tropical Agriculture (CIAT) provided data on forage digestibility and protein content [54]. We used the same procedure to estimate GHG emissions for the three farm typologies for the entire year in kg CO_2_-eq. FPCM. Table 1 summarizes the assumption factors included in the calculator to estimate GHG emissions from each particular source. The average values reported by Nicacentro were used because all the selected farmers sell their milk to this farmer cooperative.

**Table 1:** Assumptions to estimate GHG emissions.

Assumption factor	Unit	Value	Reference
GWP of CH_4_	CO_2_-eq.	21	[52]
GWP of N_2_O	CO_2_-eq.	295	[52]
Glyphosate emissions factor	kg CO_2_-eq. kg^-1^	16	[55]
2,4-D emissions factor	kg CO_2_-eq. kg^-1^	3.06	[55]
Urea N 46% emission factor	kg CO_2_-eq. kg^-1^	3.3	[55]
Maize concentrate emission factor	kg CO_2_-eq. kg^-1^	0.5	[55]
Production period for milk and gained weight	days	305	(Nicacentro 2015)
Fat content of milk	%	3.5	(Nicacentro 2015)

### Statistical analyses

All statistical analyses were performed using R (3.1.1) and analysis of variance (AoV) and Tukey's honest significant difference were applied to assess significant differences in the accumulated C between land use types, and to identify C sequestration potential. Accumulated C was log-transformed because the data did not follow a normal distribution. For GHG, we used AoV to test differences in the emissions from enteric fermentation and manure residues and between each herd category (lactating cows, dry cows, heifers, calves and bulls). The aim was to identify which animal category emits more and what was the relationship between emissions and quality and digestibility of the feed.

## Results

### Livestock production systems

About 90% of the study region is occupied by mixed crop-livestock farms. Typical crops are annuals such as maize, beans and sorghum, and perennials like coffee, cocoa and sugar cane. Grains are for domestic use, cash sale and sometimes animal feed, while plantations are cash crops. Tree legumes, such as *Gliricidia sepium* and *Guazuma ulmifolia*, are used as protein banks by 26% of farmers, while 30% use cut-and-carry forages (*Pennisetum* spp). Pruning residues are used by 73% of farmers as cattle feed, and 50% apply cattle manure as fertilizer to crops.

Although the naturalized grasses *Hyparrhenia rufa*, *Andropogon gayanus* and *Cynodon nlemfluensis* are still common, improved grasses have been widely adopted and in some farms they predominate. The most common improved grasses are *B*. *brizantha* ‘Marandú’ and ‘Toledo’ and *Panicum maximum* ‘Tanzania’ and ‘Mombasa’. All farmers have some trees in their pastures and also as living fences. Most common species are *Guazuma ulmifolia*, *Cordia alliodora*, *Gliricidia sepium*, *Lonchocarpus retiferus* and *Tabebuia roseaa*, which are used for firewood, forage, shade and construction timber. We identified 70 tree species across all systems (Table 2). There are plots of secondary forest, mean area 2 ha, on 33% of farms, corresponding to 6% of the total area.

**Table 2:** Tree density, number of tree species and carbon stocks of the five land use systems (Mean±standard error).

Land use	n*	Tree density ha^-1^	No of tree species	Pasture carbon (Mg C ha^-1^)	Tree carbon (Mg C ha^-1^)	Total carbon (pasture + tree) (Mg C ha^-1^)
NP	14	-		0.7±0.1 ab^‡^	-	0.7±10.1 a
NPT	12	86±10	32	0.4±0.1 b	24.0±5.0 a	24.4±5.1 b
IP	29	-		1.2±0.2 a	-	1.2±0.2 a
IPT	28	70±3.5	57	1.0±0.2 a	16.4±2.3 a	17.4±2.5 b
SF	9	241±11	34	2.8±0.4^†^c	43.3±13 b	46.1±13.4 c

*Number of farms.

Allometric equations applied to estimate carbon stock:

Secondary wet forest [40] Y = 0.0509 x (wood density x ((DBH)^2) x height) ^0.916))).

Secondary forest [41] log Y = -4.4661+2.707 x log (DBH).

Dispersed trees [38] log Y = -2.18062 + 0.08012 (DBH) - 0.0006244 (DBH).

^‡^ Values with different letters differ significantly (P<0.05), Tukey’s test.

^†^ Refers to litter.

Average farm size is 33±5.6 ha, of which 26±5.3 ha is used for livestock, including pasture and forage banks, 3±0.3 ha for crops, and 1.3±0.4 ha for sugarcane (Table 3). Rotational grazing is practiced by 43% of farms with an average stocking rate of 1.2 livestock units (LSU) ha^-1^. Most farms use firewood for cooking and 36% have electricity connected, although it is only used domestically. Another 36% of farmers have solar panels and 10% have biodigesters to produce gas for cooking. Engine-driven machinery, such as choppers, is used by 83% of farmers, and 66% own a motorcycle or car, of which less than half are used to transport inputs. Fertilizer is used by 60% of farmers mostly for crops.

**Table 3:** Variables defining the three livestock production systems according specific factors and GHG emissions in CO_2_-eq. (FPCM)^-1^ (n = 16).

Category	Conventional subsistence	Conventional market	Climate smart	
GHG emissions per product				
kg of CO2 –eq. (kg FCPM)^-1^	3.1	2.4	1.7	
CH_4_ enteric fermentation	2.2	1.4	1.3	
CH_4_ manure residues	0.1	0.06	0.04	
N_2_O manure residues	0.5	0.3	0.2	
N_2_O fertilizer	0.2	0.4	0.1	
CO_2_ input fabrication	0.06	0.1	0.01	
CO_2_ fossil fuel	0.04	0.1	0.02	
Farm typology				Average
Number of farms (n)	5	5	6	5
Average farm size (ha)	25 (±3.4)	53 (±15)	20 (±4.8)	33 ±(5.6)
Average areas under pasture (%)	80 (±5.7)	89 (±3.7)	77 (±1.8)	82 ±(5.6)
Other land uses	Naturalized pasture, improved pasture, annual crops, sugarcane	Improved pasture, naturalized pasture, cut and carry fodder, sugarcane, annual crops	Improved pasture, naturalized pasture, forage bank, cut and carry fodder, secondary forest, sugarcane, annual and perennial crops	
Silvopastoral system	No	No	Yes	
Grazing management	Set stocked	Rotational	Rotational	
Average herd size	28 (±5.1)	55 (±13)	24 (±6)	
Cattle breeds	Brahman, Brown Swiss crosses	Brahman, Holstein, Jersey, Brown Swiss crosses	Brahman, Holstein, Jersey, Brown Swiss crosses	
Average production level (kg milk animal^-1^day^-1^)	3.4 (±0.3)	5.9 (±0.4)	6.2 (±0.2)	
Feeding	Naturalized grasses, improved pasture, crop residues	Improved pasture, cut carry fodder, naturalized pasture	Improved pasture, cut carry fodder, forage bank, naturalized pasture	
Supplementary feeding	Mineral salt Conventional salt	ConcentrateCane molasses	Cane molasses, crop residues	
Input use	None or very low herbicides and fertilizer	High herbicides and fertilizer	Biofertilizers, manure residues	

The predominant cattle breeds are Brahman and Brown Swiss crosses, with an average herd size of 40±5.9 head. The predominant system is dual purpose, with milk generating most of the cash income. Farmers provide salt, other minerals and vitamin supplements to livestock. In general, lactating cows receive additional supplements during the dry season, including concentrate and molasses.

### Carbon stocks of livestock production systems

The SF has a significantly higher C stock than the other land uses, which is due to more woody and herbaceous biomass, including litter. Herbaceous biomass is significantly higher in IP than NP. Trees of NPT have larger DBH than IPT, and thus store more C, although differences are not significant (Table 2). Land use systems with improved grasses contain higher C stocks than their equivalents with natural grasses, whereas C stocks in SF are highest (Table 2). Although not assessed in the present study, soil C stocks did not differ significantly between land use systems in Matiguás [38]. Soil C stock to a depth of 80 cm ranged between 150–168 Mg C ha^-1^ [38].

### GHG emissions of livestock production systems

**Fig 2:**
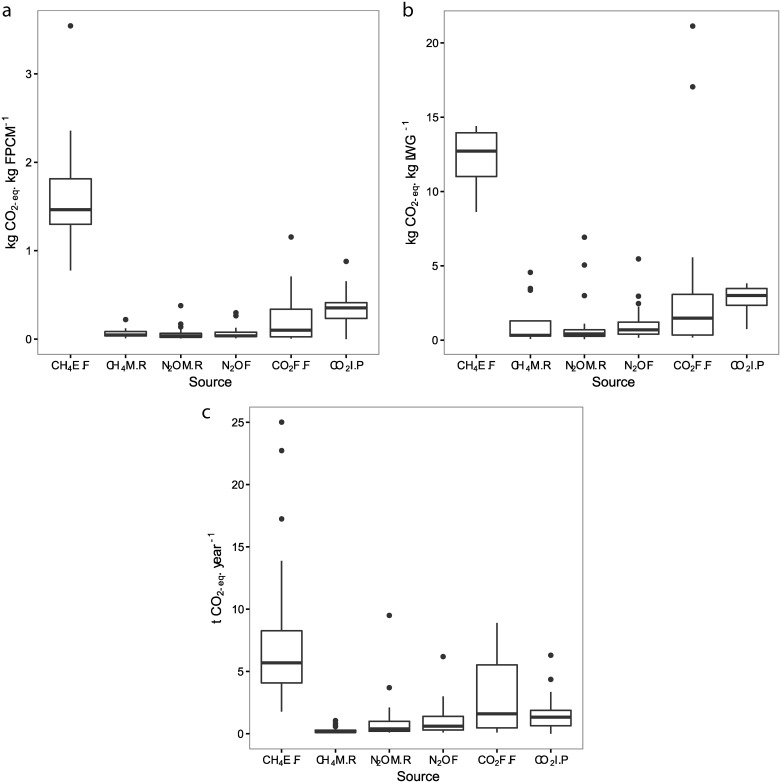
Mean GHG emissions (n = 30) from different sources in relation to **a.** milk produced; **b.** liveweight gain; or **c.** per year. The emission sources are methane from enteric fermentation (CH_4_E.F), methane from manure residues (CH_4_M.R), nitrous oxide from manure residues (N_2_OM.R), nitrous oxide from fertilizer use (N_2_OF), carbon dioxide from fossil fuel (CO_2_F.F) and carbon dioxide from producing inputs (CO_2_I.P). Emissions are expressed in **a.** kg CO_2_-eq. (kg FPCM^†^)^-1^ **b.** kg CO_2_-eq. (kg LWG)^-1^, live weight gained animal^-1^ day^-1^ **c.** tonnes CO_2_-eq. per farm year^-1^. Circles indicate outliers. ^†^FPCM = raw milk (kg) * (0.337 + 0.116 * fat% + 0.06 * protein%).

Estimates of total GHG emissions include animal digestion processes, farm operations and production of external inputs, and averaged 2.4 kg CO_2_-eq. FPCM (Fig 2a) and 28.2 kg CO_2_-eq. LWG (Fig 2b). Methane emissions from enteric fermentation account for 53–67% of the total (Fig 2), making it the major source, followed by nitrous oxide from manure (13–17%) and fertilizer (8–15%). For LWG, more nitrous oxide is emitted from fertilizer than from manure (Fig 2b). Methane from enteric fermentation and carbon dioxide from fossil fuel vary stronger between farms than other sources (Fig 2c). Carbon dioxide from fossil fuel varies greatly among farms, although on average it is not a major source of emissions.

**Fig 3:**
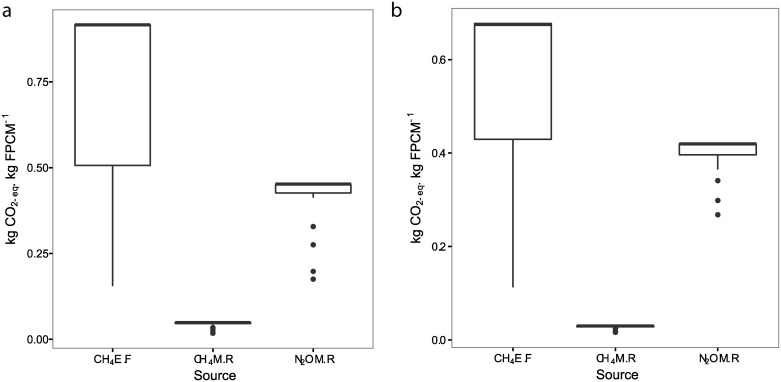
Mean GHG emissions (n = 30) for different animal categories. Sources are the sum of methane from enteric fermentation, and methane and nitrous oxide from manure. The animal categories are lactating cow, calf, heifer > 2 years (H > 2), bull, heifer 1–2 years (H1-2) and dry cow. Circles indicate outliers.

Emissions from digestion processes differ between animal categories, with lactating cows emitting most and varying least. Herd structure and production per cow therefore account for the different levels of emissions from livestock for each farm (Fig 3).

**Fig 4:**
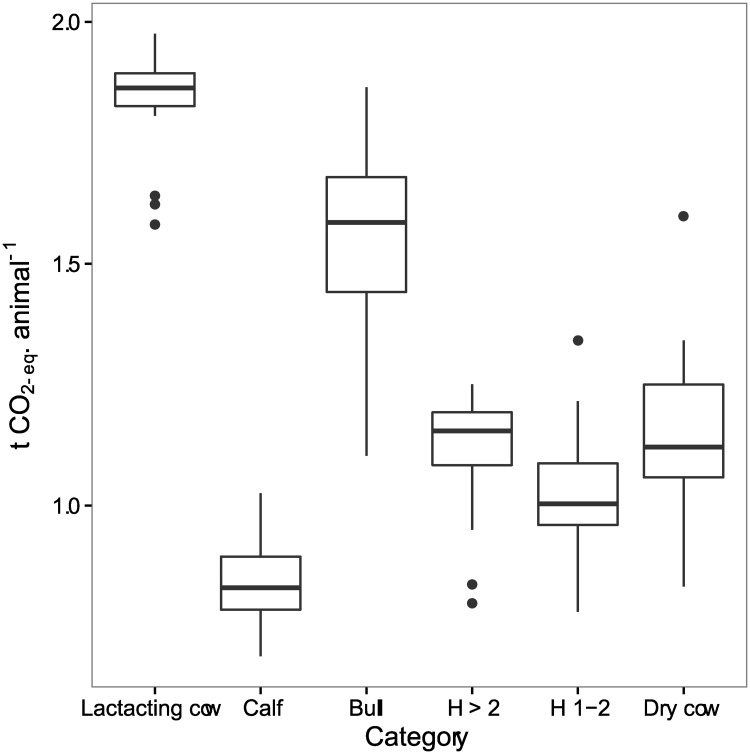
Mean GHG emissions (n = 30) from milk production in a. dry season and b. wet season. Sources are methane from enteric fermentation (CH_4_E.F), methane from manure (CH_4_M.R) and nitrous oxide from manure (N_2_OM.R).

Animal emissions are higher in the dry season than in the wet season. Enteric fermentation emits more CO_2_-eq. from methane than manure emits as methane and nitrous oxide together. Methane emissions also varies more between farms during the dry season. Methane from manure accounts for less emissions than either enteric fermentation or fertilizer. Nitrous oxide emissions vary most between farms during the wet season, with some outliers in both seasons (Fig 4).

### GHG emissions of three typical livestock production systems

As indicated before, we selected 16 small- and medium-sized farmers from among the original 30 (Table 3). We allocated them to one of three categories: subsistence system, conventional market system, and climate-smart system (allocated expost as naturally adopted). Five farmers used milk for home consumption only; five were commercial producers while the remaining six used climate-smart management. The latter seek high milk yield while managing their farms to adapt to and to mitigate climate change. They combine grazing, pasture and herd management, with feed supplements, trees and judicial application of inputs to create climate smart management. Although the exact combination is constrained by farm size it provides resilience in the face of climate change. The main emphasis is milk production, which provides 74% of farm income, the remaining 26% coming from meat [56, 57].

#### Subsistence system

These are smallholders focused at meeting livelihood needs, and milk production mainly for domestic use with only a small amount sold. Farms are about 25 ha, of which 80% is naturalized pasture although there are some improved grasses. Smallholders use crop residues, which are usually nutritionally poor quality, as cattle feed more than in the other systems. Herds average 28±5.1 Brown Swiss and Brahman crosses. Grazing is not controlled and the only supplement is mineral salts. Subsistence smallholders do not apply livestock manure and rarely use herbicides or fertilizer on their crops.

#### Conventional market system

These are commercial dairy farmers. Farms average 60 ha with 89% under improved, naturalized and cut-and-carry grasses, have little cropping and no secondary forest. Improved grasses, usually *Brachiaria* spp., have largely replaced naturalized grasses. Herds average 55±13 predominantly Brahman crosses often with Holstein and Jersey dairy breeds. Farmers use rotational grazing and supplement lactating cows during the dry season with commercial concentrates and cane molasses. They use lots of herbicides and fossil fuel, the latter to transport inputs and to chop cut-and-carry grasses.

#### Climate-smart system

Climate-smart farmers aim to increase production through climate-smart management. Farms average 20 ha, of which 77% grow forages in mixed systems. These include improved pastures with trees under rotational grazing, cut-and-carry grasses and forage trees (principally *Gliricidia sepium)*, together with some crop residues. Herds average 24±6 Brahman, Brown Swiss and Holstein crosses. The combination of well-managed improved pastures, legume trees and crop residues provides adequate feed during the dry season so that expensive feed supplements are rarely needed. Biodigesters process waste, with the residue used to fertilize forages and food crops. Secondary forest conserves natural resources and can even out stream hydrology in the dry season. Climate-smart farmers use few herbicides and little chemical fertilizer, but some use compost.

## Discussion

This study confirms previous research that well-managed livestock systems in the tropics can increase productivity, provide ecosystem services, reduce GHG emissions per kg of milk and meat, and accumulate carbon [58, 59, 60]. It provides supporting field evidence for the LivestockPlus concept, which aims to increase meat and milk production by smallholders using well-managed tropical forages and enhance the sustainability of mixed crop-forage-livestock systems. It also seeks to reduce agriculture’s carbon footprint by providing ecosystem services, including better soil quality, reduced soil erosion and reduced GHG emissions per unit of product [13].

### Carbon accumulation in well-managed mixed crop- livestock systems

C stocks of well-managed mixed crop- livestock systems are dominated by those of soils and trees, compared with which the stocks in the forages themselves are unimportant. Although the difference in C stocks have little influence on the total C stock of the system, the higher amount in IP compared to NP suggests a higher turnover of the standing material and therefore a higher contribution to soil carbon [19].

Improved pastures occupy 70% of the area of the evaluated livestock farms, of which 75% are silvopastoral systems, which emphasizes the importance of the tree component in C stocks. Chacón-León and Harvey [29] reported 13.5 Mg C ha^-1^ from dispersed trees on pastures in Nicaragua, and Amezquita et al. [27] recorded C stocks of 13.9 Mg ha^-1^ in a *B*. *brizantha* pasture with *Acacia mangium*.

Trees that were managed for regeneration in a spontaneous silvopastoral system in Ecuador did not reduce cattle productivity [61], which is an obvious advantage. A higher proportion of legume trees and shrubs in forage banks would increase soil nitrogen and nutrient uptake due to their deep root systems [62, 63]. The extra nitrogen would stimulate the grass component and increase the accumulation of soil C stocks [19].

Restoration of degraded grasslands can increase soil C. In the Colombian Llanos, SOC (to a depth of 80 cm) with *B*. *humidicola* was 223 Mg ha^-1^, and 268 Mg ha^-1^ when associated with the legume *Arachis pintoi*. Native savanna in contrast contained SOC of only 197 Mg ha^-1^. [64].

SF remains an important carbon sink in agricultural landscapes in Central America [28, 26, 38]. It also provides other ecosystem services by conserving biodiversity and retaining water, which increases ability for farmers to participate in PES [65]. Finally, well-managed pastures of improved grasses and legumes increase livestock productivity and accumulation of soil C. These systems also use less land for the same or higher animal production, sparing land for other purposes such as secondary forest [66].

### Feed quality and production efficiency as major contributors to GHG emissions

Methane from enteric fermentation is the major contributor to total GHG emissions and is directly related to the nutritional and energetic efficiency of the animal [67, 68, 69]. Digestibility and protein content of the feed are important factors that arise from seasonal differences, herd and grazing management, and supplementary feeding, among others [70]. For instance, it was reported that improved natural grass with sorghum in southern Brazil with dry matter digestibility of 52–59% resulted in lower values of CH_4_ and N_2_O emissions compared to natural grass of lower quality of 45% [60]. CIAT researchers also reported average values of dry matter digestibility of 55–62% for the two cultivars of *Brachiaria brizantha* (‘Toledo’ and ‘Marandu’);due to higher digestibility than the 50% rate of the naturalized grass *Paspalum notatum* they could reduce CH4 and N2O emissions per unit livestock product.[51]. Considering the fact that lactating cows emit more CH_4_ compared to other animal categories, feeding those cows with improved forages of higher digestibility would decrease CH_4_ emissions, and would be also more profitable for the famer. We estimate higher emissions during the dry season due to lower availability of feed of good quality. The subsistence farmers use only low quality crop residues, which sharply reduces milk yield and causes the cows to lose weight [57]. This explains the large variation among farms in terms of CH_4_ emissions from enteric fermentation during the dry season (Fig 3).

Manure emits proportionally more N_2_O because it is not spread on the pastures but left in the corrals where nitrogen is leached and volatilized [71]. Carbon dioxide contributes least to GHG emissions from livestock, because the systems use few inputs and little machinery and transport.

Latin American countries mainly rely on sown pastures for year-round production [72]. In some regions deforestation for establishing cattle pastures is a major source of CO_2_ emissions [73, 9, 74]. We have not included that aspect here as the land was cleared many decades ago.

FAO estimated 2.4 kg CO_2_ -eq. FPCM^-1^ as the global average, which is similar to the present study, and 3.2 kg CO_2_ -eq. FPCM^-1^ for LCA [69]. However, they included activities after the farm gate and emissions originating from land conversion, which we did not consider.

### Well-managed mixed crop-livestock systems reduce GHG emissions

The climate-smart farms increased milk production while reducing GHG emissions and increasing carbon stocks. Higher-quality feed with higher digestibility, protein and energy content produced less methane per unit of animal product [21]. A further option for the future is the use of methane inhibitors [75], which appear to be successful, but would need to be tested in the tropics. Leguminous forages produce high-quality feed and reduce methane emissions, because they contain more condensed tannins which increase the absorption of essential amino acids [76, 77]. Cost-effective supplementary feed for the dry season are sugarcane and legume protein banks in cut-and-carry systems, but they require more investment than many farmers can afford [57].

Subsistence farming has low productivity mainly due to low feed quality, especially during the dry season. Protein and energy intake are low, mostly at maintenance level, which gives high emissions per unit of product [57, 69]. Furthermore, poor farmers have little control over diseases and parasites and their cattle have low genetic potential leading to low fertility, high mortality and low growth rates [78]. Their farms are located in areas that lack basic infrastructure.

Market-oriented and climate-smart systems have access to feed of higher quality (Table 3) leading to higher milk yield. Cows in silvopastoral systems of Nicaragua were shown to produce 5.9 kg day^-1^ compared with only 3.8 kg day^-1^ in traditional systems [79]. As productivity increases, farmers can reduce herd size to produce the same amount or more [80, 81, 69, 67].

Subsistence-oriented and market-oriented systems had highest emissions of N_2_O, mainly from inadequate manure management. Higher-quality feeds and efficient manure management are valid mitigation options for reducing emissions. It is known that 80% of the nitrogen ingested by the animal is excreted [82, 83], and applying that as manure to crops and pastures serves as fertilizer and reduces emissions. Moreover, anaerobic digesters on climate-smart farms convert manure into CH_4_ used as fuel (for cooking, but can also be used for cooling of milk). The residue is used as a fertilizer [41]. *B*. *humidicola* produces biological nitrification inhibitors (BNIs) in soil, which also reduces N_2_O emissions [24, 84, 85].

Improved grazing management optimizes productivity and offers mitigation and adaptation benefits [16]. In this context proper implementation of rotational grazing is suggested to optimize the availability of good quality grass biomass in relation to animal requirements, leading to improved productivity, increased carbon accumulation in soil and reduced land degradation.

Climate-smart systems are being implemented, albeit still at a small scale. Improved forage-based systems are not adopted by subsistence- and market-oriented farmers because there are no incentives, whereas policies are often inadequate or not enforced. CIAT and its partners are implementing the LivestockPlus concept in four regions of Colombia and two regions of Costa Rica. The objective is to stimulate nationally appropriate mitigation actions (NAMAs), implement pasture management that emits less GHGs and assess the socioeconomic impact as part of nationally determined contributions (NDCs). A further objective is to generate the best mitigation options and low-cost methods to quantify GHG emissions [72]. The private sector is also starting to reduce their carbon footprint by investing in carbon credits. For smallholders the challenge is not only to sustain farms that provide livelihoods and environmental benefits, but also how to consolidate and sell the products [32].

## Conclusions

Livestock production systems in Central America can reduce their carbon footprint by improving productivity and realizing social, economic and environmental benefits.

Well-managed mixed crop-livestock systems based on forages increase the quality of animal feed and reduce methane and N_2_O emissions, particularly from enteric fermentation and adequate manure management. Carbon dioxide was not a major component of the emissions of the farms in the present study. Nevertheless, land-use conversion was historically a main contributor in LAC and should be accounted in any further studies.

Growing trees in pastures increases the capacity of the system to accumulate C; and adequate densities of sizable trees should be advocated. Secondary forest accounts for the highest C stocks, and should be considered as a vital farm component to provide different ecosystem services. Farms that were climate-smart had higher milk yields per animal, allowing farmers to intensify the system through reduced herd size and pasture area. Although promotions already increased the number of climate-smart farms, large-scale implementation will depend on adequate policies with effective incentives. In this regard, the private sector is becoming aware of the opportunities to reduce their carbon footprint by investing in carbon credits. Smallholders who implement sustainable production systems could play an important role in the sustainable intensification of livestock systems.

## Supporting Information

S1 Fig Study site at Matiguás, Nicaragua. Location of the 30 farms, of which 16 were classified as one of the three typologies: conventional subsistence, conventional market and climate smart. Reprinted from [39] under a CC BY license, with permission from [CGIAR-CSI], original copyright [2008].
(XLSX)

S2 Fig Mean GHG emissions (n = 30) from different sources in relation to milk. The emission sources are methane from enteric fermentation (CH_4_E.F), methane from manure residues (CH_4_M.R), nitrous oxide from manure residues (N_2_OM.R), nitrous oxide from fertilizer use (N_2_OF), carbon dioxide from fossil fuel (CO_2_F.F) and carbon dioxide from producing inputs (CO_2_I.P). Emissions are expressed in kg CO_2_-eq. (kg FPCM^†^)^-1^.Circles indicate outliers. ^†^FPCM = raw milk (kg) * (0.337 + 0.116 * fat% + 0.06 * protein%).
(CSV)

S3 Fig Mean GHG emissions (n = 30) from different sources in relation to liveweight. The emission sources are methane from enteric fermentation (CH_4_E.F), methane from manure residues (CH_4_M.R), nitrous oxide from manure residues (N_2_OM.R), nitrous oxide from fertilizer use (N_2_OF), carbon dioxide from fossil fuel (CO_2_F.F) and carbon dioxide from producing inputs (CO_2_I.P). Emissions are expressed in kg CO_2_-eq. (kg LWG)^-1^, live weight gained animal^-1^ day^-1^. Circles indicate outliers.
(CSV)

S4 Fig Mean GHG emissions (n = 30) from different sources per year. The emission sources are methane from enteric fermentation (CH_4_E.F), methane from manure residues (CH_4_M.R), nitrous oxide from manure residues (N_2_OM.R), nitrous oxide from fertilizer use (N_2_OF), carbon dioxide from fossil fuel (CO_2_F.F) and carbon dioxide from producing inputs (CO_2_I.P). Emissions are expressed in tonnes CO_2_-eq. per farm year^-1^. Circles indicate outliers.
(CSV)

S5 Fig Mean GHG emissions (n = 30) for different animal categories. Sources are the sum of methane from enteric fermentation, and methane and nitrous oxide from manure. The animal categories are lactating cow, calf, heifer > 2 years (H > 2), bull, heifer 1–2 years (H1-2) and dry cow. Circles indicate outliers.
(XLSX)

S6 Fig Mean GHG emissions (n = 30) from milk production in dry season. Sources are methane from enteric fermentation (CH_4_E.F), methane from manure (CH_4_M.R) and nitrous oxide from manure (N_2_OM.R).
(CSV)

S7 Fig Mean GHG emissions (n = 30) from milk production in wet season. Sources are methane from enteric fermentation (CH_4_E.F), methane from manure (CH_4_M.R) and nitrous oxide from manure (N_2_OM.R).
(CSV)

S1 Table Tree density, number of tree species and carbon stocks of the five land use systems (Mean±standard error). Accumulated carbon in pasture and trees on the five land use systems expressed in Mg C ha^-1^.
(XLSX)
